# Correlation Analysis of Neutrophil/Albumin Ratio and Leukocyte Count/Albumin Ratio with Ischemic Stroke Severity

**DOI:** 10.26502/fccm.92920305

**Published:** 2023-02-13

**Authors:** Sanying Mao, Yuanhong Hu, Xingwu Zheng, Chengmin Yang, Meiling Yang, Xianghong Li, Jingwei Shang, Koji Abe

**Affiliations:** 1 Department of Neurology, The First People's Hospital of Jiande, Hangzhou, China; 2 Guilin Medical University, Guilin, China; 3 Department of Neurology, Affiliated Hospital of Youjiang Medical University for Nationalities, Baise, China; 4 National Center of Neurology and Psychiatry (NCNP), Kodaira city, Tokyo, Japan

**Keywords:** Albumin, Ischemic Stroke, Leukocyte Count/Albumin Ratio, Neutrophil/Albumin Ratio

## Abstract

Ischemic stroke (IS) is a common neurological disease in the elderly, but the relationship between neutrophil/albumin ratio (NAR) and leukocyte count/albumin ratio (LAR) and the severity of neurological function injury and early neurological deterioration (END) occurrence remain elusive in acute IS. A total of 299 patients with acute IS and 56 healthy controls were enrolled. According to the NIHSS score at admission, the disease group was divided into three groups (mild, moderate and severe IS), and the differences in five indexes NAR, LAR, neutrophil count, leukocyte count and albumin among the four groups were analyzed. Furthermore, explore the correlation between the above indicators and the severity of IS and END occurrence. The results showed that higher NAR, LAR, neutrophil count, leukocyte count levels and lower albumin levels were associated with acute IS, and the levels of NAR and LAR increased gradually in three groups of IS. NAR and LAR were positively and albumin was negatively correlated with the severity of IS. Meanwhile, NAR and LAR showed a good predictive value in identifying patients with END after acute IS. NAR and LAR may be predictors of the severity of IS and END occurrence after acute IS.

## 1. Introduction

Ischemic stroke (IS) is a common neurological disease in the middle-aged and elderly population with high morbidity, mortality, and disability rate [1]. In China, IS has an annual death rate of about 1.6 million and a mortality rate of about 157/100,000, which is higher than that of cardiovascular disease and has become one of the main causes of death and disability in adults [2]. Rapid assessment of the severity of IS in clinical treatment and individualized treatment may play an important role in preventing the deterioration of the patien's condition, reducing complications, and reducing the incidence of mortality and disability. Atherosclerosis is a risk factor for IS, the early formation of atherosclerosis is associated with the accumulation of leukocytes and proinflammatory cytokines. So, atherosclerosis development is accompanied by an inflammatory reaction, which accelerates thrombosis formation and promotes the occurrence of IS and myocardial infarction [3,4]. Serum leukocyte detection is a common, inexpensive and simple indicator of inflammation in clinical work, including neutrophils, monocytes and other sub-cells. The mechanisms of leukocyte sub-cells in the process of acute IS are different. Among them, neutrophils converge in the ischemic penumbra area and release proteolytic enzymes, which damage the blood-brain barrier and promote the accumulation of inflammatory factors in the ischemic area, aggravating brain tissue damage [5,6]. Albumin plays important role in maintaining colloid osmotic pressure balance and influencing microvascular integrity and inflammatory pathways, including neutrophil adhesion [7]. Some studies have shown that the neurological impairment in IS patients may be related to the decreased protection of albumin on ischemic brain tissue [8], and the serum albumin level has a predictive role in the prognosis of acute IS [9]. The neutrophil/albumin ratio (NAR) is a comprehensive inflammation biomarker, which has been used in many clinical studies. For example, in the association study between NAR and delayed cerebral ischemia (DCI) in the early stage of aneurysmal subarachnoid hemorrhage (aSAH), NAR was found to be positively correlated with the severity of subarachnoid hemorrhage, which can be used as a new predictive biomarker for DCI after aSAH [10]. NAR is a potential prognostic biomarker for mortality in patients with cardiogenic shock (CS), and its predictive value is more sensitive than neutrophil percentage or serum albumin level alone [11]. At present, there is no study on the correlation between NAR and the severity of acute IS and early neurological deterioration (END) occurrence after acute IS. Therefore, we performed a retrospective cohort study to analyze the expressive changes of five accessible and inexpensive indexes NAR, leukocyte count/albumin ratio (LAR), albumin, neutrophil count and leukocyte count in patients with acute IS. To determine the relationship between the five indexes and the severity of neurological function injury and END occurrence within acute IS, to better timely treatment of IS and reduce mortality and improve the prognosis.

## 2. Materials and Methods

### 2.1 Study Population

This was a retrospective, observational study. 299 consecutive patients with acute IS were enrolled from November 2016 to October 2021 at the Affiliated Hospital of Guilin Medical College. 56 age- and sex-matched individuals with no neurological or psychiatric diseases found in medical examinations were included as normal controls. This study was approved by the Ethics Review Committee of Affiliated Hospital of Guilin Medical College and consent was obtained from all participants prior to enrollment. The inclusion criteria were as follows: (1) hospital admission within 48 hours of first stroke onset; (2) symptoms consistent with the 2014 Chinese Acute Ischemic Stroke Diagnostic Criteria with the responsible lesions identified via DWI [12]; (3) the age of onset is more than 18 years old; (4) National Institutes of Health Stroke Scale (NIHSS) score [12] measured within 24 hours after admission; (5) detection of blood biochemical indicators within 24 hours of admission; (6) the score of WORSEN was calculated according to Miyamoto et al [13], and (7) early neurological deterioration (END) was defined as an increase in two or more NIHSS points, an increment of at least one point in motor power, or description of fluctuating of clinical symptoms in medical reports during the first 7 days after admission [14]. The exclusion criteria were as follows: (1) hemorrhagic stroke, transient ischemic attack (TIA), multiple sclerosis (MS), intracranial infection or other diseases; (2) patients who accepted intravenous thrombolysis and(or) mechanical thrombectomy; (3) previous history of IS, TIA, cerebral hemorrhage, serious infection, major surgery, or more severe trauma; (4) severe heart, liver, kidney, lung, digestive tract and other important organ damage, blood, immune diseases and tumors. These patients take many types of medications, which may have adverse factors affecting the inflammatory response; (5) history of major trauma, surgery, blood transfusion, blood donation or immunization in the past six months; (6) pregnant women, dementia, persons with neurological disabilities, and persons with severe psychological disorders; (7) Treatment with hormone drugs, immunosuppressive drugs, or nonsteroidal anti-inflammatory drugs before or after the onset. The drugs have an impact on the immune response and inflammatory response in the body; (8) pneumonia, bloodstream infection, urinary infection, infectious diarrhea, catheter-related infection, and/or sinusitis; (9) early discharge and/or incomplete clinical data.

### 2.2 Risk Factor Assessments

The related risk factors considered in the present investigation were summarized in Table 1, hypertension: an arterial systolic blood pressure ≥140 mmHg and/or arterial diastolic blood pressure ≥ 90mmHg without the use of blood pressure-lowering drugs measured twice by continuous monitoring of arterial blood pressure [15]. A previous diagnosis of hypertension and continued use of the relevant drugs to control blood pressure were also used to define hypertension; diabetes: random venous blood glucose > 11.1 mmol/L, fasting blood glucose > 7.0 mmol/L, or OGTT 2-h blood glucose > 11.1 mmol/L and accompanying symptoms of diabetes [16]. A previous diagnosis of diabetes and the current use of blood glucose control drugs were also used to define diabetes; dyslipidemia: (1) total cholesterol (TC) ≥ 6.22 mmo1/L, (2) triglyceride (TG) ≥ 2.26 mmo1/L, or (3) low-density lipoprotein (LDL-C) ≥ 4.14 mmo1/L [17]; smoking: (1) previous or current regular smoking habit and smoking > 10 cigarettes/day and (2) smoking duration > 1 year or smoking cessation < 10 years [18], and drinking: daily consumption of alcohol with an average alcohol intake > 50 g/day [19].

### 2.3 Laboratory Measurements

The clinical information on the normal controls and the patients is summarized in Table 2. According to the NIHSS score, the observation group was divided into a mild IS group (1≤NIHSS score≤4), moderate IS group (5≤NIHSS score≤15), severe IS group (16≤NIHSS score). Fasting blood samples were collected within 24 hours after admission and were measured at the Affiliated Hospital of Guilin Medical College by using an automated analytical platform (Beckman Coulter AU5800: Beckman Coulter Inc. Brea, CA, USA). HDL-C, LDL-C, TC, TG and albumin were measured using blood samples drawn at approximately 7 a.m. after an overnight fast. White blood cell count, neutrophil count and lymphocyte count in EDTA-anticoagulated whole-blood samples from venipuncture were determined with automated particle counters within the first 24 h after admission. NAR and LAR were calculated as the ratio of neutrophil count to albumin and leukocyte count to albumin.

### 2.4 Statistical Analysis

Statistical analyses were performed using standard statistical software (SPSS 22.0, IBM Corp., Armonk, NY, USA). The measurement data were represented by (x ± s). Logistic regression analysis was performed to evaluate the related risk factors on IS, adjusting for baseline variables when a *p*< 0.1 was found in the univariate analysis. One-way ANOVA and Kruskal-Wallis tests were performed to analyze the differences among four groups followed by the Kolmogorov-Smirnov test. Pearson's correlation analysis was performed to analyze the Correlation analysis between the levels of the neutrophil count, albumin, NAR, leukocyte count and LAR and the NIHSS score. We calculated the sensitivity and specificity of different levels of NIHSS and WORSEN scores, NAR and LAR for the prediction of END by using receiver operating characteristic (ROC) curves. Differences with a probability value of *p*<0.05 were considered statistically significant.

## 3. Results

### 3.1 Logistic Analysis of the related Risk Factors of Acute IS

299 acute IS patients and 56 normal controls were included in this study. The results of the logistic analysis showed that gender (*p*<0.05, OR=0.334), smoking (*p*<0.01, OR=0.161), drinking (*p*<0.01, OR=11.653), diabetes mellitus (DM, *p*<0.01, OR=0.099), hypertension (*p*<0.01, OR=0.051), and coronary heart disease (CHD, *p*<0.05, OR=10.360) were all related to the onset of IS (Table 1).

### 3.2 Laboratory Indicators Analysis

IS patients were evaluated according to NIHSS score, there were 100 cases in the mild IS group (1 point≤NIHSS score≤4 points), 81 cases in the moderate IS group (5 points≤NIHSS score≤15 points), and 23 cases in the severe IS group (16 points≤NIHSS score). The results of laboratory indicators were presented in Table 2. Compared with the normal controls, the levels of the neutrophil count, neutrophil ratio, leukocyte count and lymphocyte count were significantly higher, meanwhile, albumin levels showed significantly lower in the three IS groups, but no significant differences were found among them. The levels of NAR, LAR, NIHSS score and WORSEN score were remarkably higher than in the normal controls, meanwhile, with a significant difference among the three IS groups. About the results of serum lipid examination, only the level of HDL-C was remarkably lower in the three IS groups than in the normal controls.

### 3.3 Correlation Analysis between the Levels of the NAR, LAR, Albumin, Neutrophil Count and Leukocyte Count and the NIHSS Score

**Figure 1:**
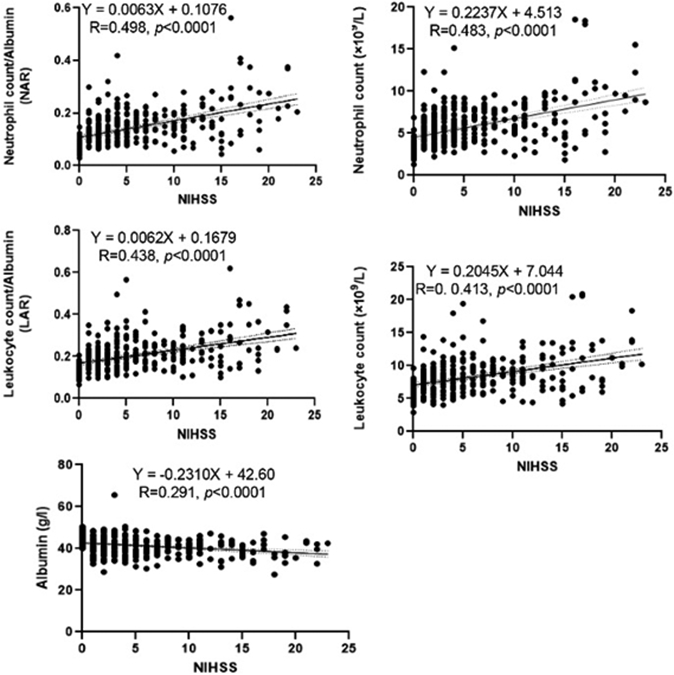
Correlation analysis between the levels of neutrophil count, albumin, NAR, leukocyte count and LAR and the NIHSS score.

The correlation analysis showed that the five indexes were significantly correlated with the NIHSS score (Figure 1), the levels of neutrophil count (R=0.483, *p*<0.0001), NAR (R=0.498, *p*<0.0001), leukocyte count (R=0.413, *p*<0.0001) and LAR (R=0.438, *p*<0.0001) were positively correlated with the NIHSS score. On the other hand, the level of albumin was negatively correlated with the NIHSS score (R=0.291, *p*<0.0001).

### 3.4 Predictive Value of WORSEN score, NIHSS score, NAR and LAR for Early Neurological Deterioration (END) using ROC

**Figure 2:**
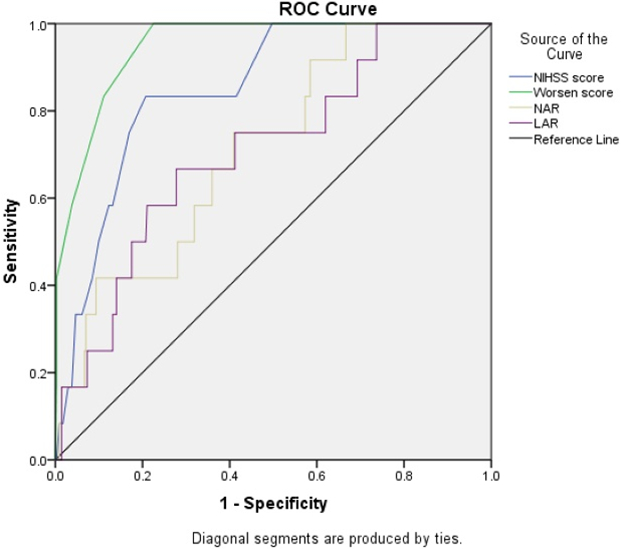
The predictive value of WORSEN score, NIHSS score, NAR and LAR for END using ROC.

In Figure 2, the area under the ROC curve (AUC) of the WORSEN score for the prediction of END was 0.95 (95%CI 0.91-0.98). The AUC of the NIHSS score for the prediction of END was 0.85 (95%CI 0.76-0.94). Meanwhile, The AUC of the NAR and LAR for the prediction of END was 0.71 (95%CI 0.58-0.85) and 0.71 (95%CI 0.56-0.85), respectively.

## 4. Discussion

Many clinical studies have focused on how to find effective, easily available and inexpensive blood-based biomarkers for the early prediction of IS severity. In this study, we examined differences in the expression levels of several plasma biomarkers in the mild, moderate and severe IS groups, and found the expressive differences are indicative of the diverse pathological mechanisms underlying IS. Our results showed that gender, smoking, drinking, DM, hypertension, and CHD were related to the onset of IS (Table 1). The results of laboratory indicators showed that the levels of the NAR, LAR, neutrophil count, leukocyte count, NIHSS score and WORSEN score were remarkably higher, and albumin remarkably lower in the three IS groups. Meanwhile, NAR, LAR, NIHSS score and WORSEN score showed significant differences among the three IS groups (Table 2). Moreover, the correlation analysis showed that the five indexes (NAR, LAR, albumin, neutrophil count and leukocyte count) were significantly correlated with the NIHSS score (Figure 1). WORSEN score, NIHSS score, NAR and LAR showed a good predictive value in identifying patients with END after acute IS (Figure 2). After IS, the levels of serum leukocytes and neutrophils increased (Table 2), because within 1-6 hours of the onset of acute IS, a large number of leukocytes, main neutrophils, adhere to the post-capillary venules and capillary walls of ischemic tissue. At the same time, oxidative damage and proteolysis of vascular endothelial cells promote the aggregation of leukocytes and red blood cells around ischemic foci, and aggravate the microcirculation disorder and blood hypercoagulability, and further reduce cerebral blood flow [20,21]. Then, over the next 6 to 24 hours, these neutrophils migrate from the damaged vessel wall to the ischemic cortical area. Infiltrating leukocytes and resident brain cells, including neurons and glial cells, release proinflammatory mediators such as cytokines, chemokines, and oxygen-nitrogen free radicals, exacerbating the evolution of brain tissue damage and leading to increased mortality [22-24]. Some studies have found that leukocyte count can be used as independent predictors of IS [25]. Our results further suggested that the level increases of NAR and LAR were associated with different severity of IS (Table 2, Figure 1). Serum albumin offers neuroprotective effects through antagonizing thrombosis, stagnation and leukocyte adhesion within the postcapillary microcirculation in the early reperfusion phase of stroke [26]. Some studies reported that the level of serum albumin is closely related to the occurrence and development of IS [27, 28]. Our result also showed that the albumin levels were significantly lower in the three IS groups, especially in the severe IS group (Table 2, Figure 1). Which suggested that the serum albumin level changes may be used to assess the severity of acute IS in elderly patients. Moreover, albumin treatment has been found to improve neurological function in rats with focal IS and clinical IS patients [29,30]. In acute IS patients, END not only has a high incidence of 5-40% [31], but is also associated with poor prognosis [32]. Our results showed that the initial NIHSS score and WORSEN score had good predictive values for END (Figure 2), which were consistent with previous studies [14]. We also found that LAR and NAR had good predictive values for END (Figure 2). Thus, inflammation may be one of the risks of END in patients with acute IS.

## 5. Limitations

This study was subject to several limitations. First, this study had a retrospective case-control design and was performed within a small area of China. Second, relatively few patients and controls were included, especially in the severe IS groups, so the validity of our results remains to be tested. Finally, no equal numbers of males and females were achieved in the study.

## 6. Conclusions

In summary, the present study is the first to analyze the changes of NAR, LAR, albumin, neutrophil count and leukocyte count levels in patients with acute IS, and found that the NAR and LAR levels were correlated with the severity of IS. Attention should be paid to the condition changes of IS hospitalized patients with high NAR, LAR, neutrophil count and leukocyte count levels but low albumin level, hoping that the prognosis can be improved by early active and effective treatment. Effective blood markers and NIHSS score and WORSEN score for IS may occur during the END provides the clinical basis, which helps to improve the treatment of acute IS clinical decision. This study further explored the early assessment of the severity of IS and END occurrence, and provided a clinical reference for large-scale marker screening and combination.

## Figures and Tables

**Table 1:** Logistic analysis of the related risk factors of acute IS. *p<0.05, **p<0.01. AF: Atrial fibrillation, CHD: Coronary heart disease, DM: diabetes mellitus, IS: ischemic stroke.

Patient characteristics	Normal	IS	OR	95% C.I.		*p*
	(n=56)	(n=299)		Lower	Upper	
Males [n(%)]	29 ( 51.78 )	224 ( 74.92 )	0.334	0.123	0.909	0.032*
Smoking [n(%)]	19 ( 33.93 )	138 ( 46.15 )	0.161	0.045	0.576	0.005**
Drinking [n(%)]	22 ( 39.29 )	74 ( 24.75 )	11.653	3.273	41.482	0.000**
DM [n(%)]	2 ( 3.57 )	68 ( 22.74 )	0.099	0.02	0.479	0.004**
Hypertension [n(%)]	9 ( 16.07 )	227 ( 75.92 )	0.051	0.021	0.125	0.000**
AF [n(%)]	0	31 ( 10.37 )	0	0	0	0.997
CHD [n(%)]	3 ( 5.35 )	7 ( 2.34 )	10.36	1.668	64.334	0.012*

AF- Atrial fibrillation; CHD- Coronary heart disease; DM- diabetes mellitus; IS- ischemic stroke

* p<0.05

** p<0.01.

**Table 2:** Comparison of laboratory indicators among each group. ^a^*p*<0.05 vs. normal, ^b^*p*<0.05 vs. mild IS, ^c^*p*<0.05 vs. moderate IS. HDL-C: High-density lipoprotein cholesterol, IS: ischemic stroke, LDL-C: low-density lipid-cholesterol.

Clinical data	Normal (n=56)	Mild IS (n=147)	Moderate IS (n=127)	Severe IS (n=25)	*P*
Age at examination (years)	66.09±7.95	65.35±11.27	67.65±11.22	73.56±8.74^ab^	0.007
Neutrophil count (×10^9^/L)	3.34±1.02	5.59±1.71^a^	6.18±1.92^a^	9.67±4.37^abc^	0
Albumin (g/l)	45.11±2.46	41.38±4.88^a^	40.15±3.68^a^	38.81±4.67^ab^	0
Neutrophil count/Albumin (NAR)	0.074±0.022	0.138±0.047^a^	0.156±0.052^ab^	0.253±0.116^abc^	0
Neutrophil ratio	0.55±0.08	0.72±0.09^a^	0.73±0.11^a^	0.82±0.11^abc^	0
Leukocyte count (×10^9^/L)	6.07±1.29	7.93±2.01^a^	8.67±2.49^a^	11.61±4.62^abc^	0
Leukocyte count/Albumin (LAR)	0.135±0.029	0.195±0.060^a^	0.219±0.071^ab^	0.303±0.122^abc^	0
Lymphocyte count (×10^9^/L)	2.10±0.62	1.45±0.56^a^	1.60±0.75^a^	1.44±1.19^a^	0
Triglycerides (mmol/L)	1.49±0.97	1.60±1.02	1.45±0.97	1.30±0.60	0.419
Cholesterol (mmol/L)	4.73±0.83	4.43±1.08	4.56±1.00	4.10±0.93	0.04
HDL-C (mmol/L)	1.40±0.38	1.19±0.33^a^	1.18±0.31^a^	1.15±0.32^a^	0
LDL-C (mmol/L)	2.91±0.72	2.99±0.98	3.13±0.94	2.75±0.89	0.188
NIHSS score	0	2.40±1.09^a^	8.97±3.21^ab^	18.60±2.20^abc^	0
WORSEN score	0	1.11±0.87^a^	2.31±1.54^ab^	4.24±1.36^abc^	0

HDL-C- High-Density Lipoprotein Cholesterol; IS- Ischemic Stroke; LDL-C- Low-Density Lipoprotein Cholesterol

a *p*<0.05 vs. normal

b *p*<0.05 vs. mild IS

c *p*<0.05 vs. moderate IS.

## References

[1] Lloyd-Jones D, Adams R, Carnethon M, Heart disease and stroke statistics--2009 update: a report from the American Heart Association Statistics Committee and Stroke Statistics Subcommittee. Circulation 119 (2009): 480–486. 19171871 10.1161/CIRCULATIONAHA.108.191259

[2] Liu L, Wang D, Wong KS, Stroke and stroke care in China: huge burden, significant workload, and a national priority. Stroke 42 (2011): 3651–3654. 22052510 10.1161/STROKEAHA.111.635755

[3] Bjorkegren JLM, Lusis AJ. Atherosclerosis: Recent developments. Cell 185 (2022): 1630–1645. 35504280 10.1016/j.cell.2022.04.004 PMC9119695

[4] Libby P. Inflammation in atherosclerosis. Nature 420 (2002): 868–874. 12490960 10.1038/nature01323

[5] Emsley HC, Smith CJ, Gavin CM, An early and sustained peripheral inflammatory response in acute ischaemic stroke: relationships with infection and atherosclerosis. J Neuroimmunol 139 (2003): 93–101. 12799026 10.1016/s0165-5728(03)00134-6

[6] Hartl R, Schurer L, Schmid-Schonbein GW, Experimental antileukocyte interventions in cerebral ischemia. J Cereb Blood Flow Metab 16 (1996): 1108–1119. 8898682 10.1097/00004647-199611000-00004

[7] Quinlan GJ, Martin GS, Evans TW. Albumin: biochemical properties and therapeutic potential. Hepatology 41 (2005): 1211–1219. 15915465 10.1002/hep.20720

[8] Alvarez-Perez FJ, Castelo-Branco M, Alvarez-Sabin J. Albumin level and stroke. The potential association between lower albumin level and cardioembolic aetiology. Int J Neurosci 121 (2011): 25–32. 20954836 10.3109/00207454.2010.523134

[9] Babu MS, Kaul S, Dadheech S, Serum albumin levels in ischemic stroke and its subtypes: correlation with clinical outcome. Nutrition 29 (2013): 872–875. 23422540 10.1016/j.nut.2012.12.015

[10] Zhang X, Liu Y, Zhang S, Neutrophil-to-Albumin Ratio as a Biomarker of Delayed Cerebral Ischemia After Aneurysmal Subarachnoid Hemorrhage. World Neurosurg 147 (2021): e453–e458. 33373740 10.1016/j.wneu.2020.12.084

[11] Peng Y, Xue Y, Wang J, Association between neutrophil-to-albumin ratio and mortality in patients with cardiogenic shock: a retrospective cohort study. BMJ Open 10 (2020): e039860. 10.1136/bmjopen-2020-039860 PMC7574943 33077569

[12] Kwah LK, Diong J. National Institutes of Health Stroke Scale (NIHSS). J Physiother 60 (2014): 61. 24856948 10.1016/j.jphys.2013.12.012

[13] Miyamoto N, Tanaka R, Ueno Y, Analysis of the Usefulness of the WORSEN Score for Predicting the Deterioration of Acute Ischemic Stroke. J Stroke Cerebrovasc Dis 26 (2017): 2834–2839. 28784279 10.1016/j.jstrokecerebrovasdis.2017.07.005

[14] Xu Y, Chen Y, Chen R, External Validation of the WORSEN Score for Prediction the Deterioration of Acute Ischemic Stroke in a Chinese Population. Front Neurol 11 (2020): 482. 32547483 10.3389/fneur.2020.00482 PMC7272667

[15] Committee of Cardio-Cerebro-Vascular Diseases of Gerontological Society of C, Chinese College of Cardiovascular Physicians of Chinese Medical Doctor A. Chinese expert consensus on the diagnosis and treatment of hypertension in the elderly(2017). Zhonghua Nei Ke Za Zhi 56 (2017): 885–893.29136726

[16] Jia W, Weng J, Zhu D, Standards of medical care for type 2 diabetes in China 2019. Diabetes Metab Res Rev 35 (2019): e3158. 30908791 10.1002/dmrr.3158

[17] Zhao SP. Amendment of the low-density lipoprotein cholesterol target in the 'Chinese Guidelines for the Prevention and Treatment of Adult Dyslipidemia': Opinion. Chronic Dis Transl Med 2 (2016): 7–9. 29063018 10.1016/j.cdtm.2016.04.001 PMC5643599

[18] Kawle AP, Nayak AR, Lande NH, Comparative evaluation of risk factors, outcome and biomarker levels in young and old acute ischemic stroke patients. Ann Neurosci 22 (2015): 70–77. 26130910 10.5214/ans.0972.7531.220204 PMC4480259

[19] Renna R, Pilato F, Profice P, Risk factor and etiology analysis of ischemic stroke in young adult patients. J Stroke Cerebrovasc Dis 23 (2014): e221–227. 24418315 10.1016/j.jstrokecerebrovasdis.2013.10.008

[20] Barone FC, Feuerstein GZ. Inflammatory mediators and stroke: new opportunities for novel therapeutics. J Cereb Blood Flow Metab 19 (1999): 819–834. 10458589 10.1097/00004647-199908000-00001

[21] Lo EH, Moskowitz MA, Jacobs TP. Exciting, radical, suicidal: how brain cells die after stroke. Stroke 36 (2005): 189–192. 15637315 10.1161/01.STR.0000153069.96296.fd

[22] Amantea D, Nappi G, Bernardi G, Post-ischemic brain damage: pathophysiology and role of inflammatory mediators. FEBS J 276 (2009): 13–26. 19087196 10.1111/j.1742-4658.2008.06766.x

[23] Kuznik BI, Morozova I, Rodnina OS, Leukocytosis and outcomes of acute stroke. Zh Nevrol Psikhiatr Im S S Korsakova 110 (2010): 10–14. 20517219

[24] Boehme AK, Kumar AD, Lyerly MJ, Persistent leukocytosis-is this a persistent problem for patients with acute ischemic stroke? J Stroke Cerebrovasc Dis 23 (2014): 1939–1943. 24784010 10.1016/j.jstrokecerebrovasdis.2014.02.004 PMC5032654

[25] Grau AJ, Boddy AW, Dukovic DA, Leukocyte count as an independent predictor of recurrent ischemic events. Stroke 35 (2004): 1147–1152. 15017013 10.1161/01.STR.0000124122.71702.64

[26] Idicula TT, Waje-Andreassen U, Brogger J, Serum albumin in ischemic stroke patients: the higher the better. The Bergen Stroke Study. Cerebrovasc Dis 28 (2009): 13–17. 19420917 10.1159/000215938

[27] Dziedzic T, Pera J, Klimkowicz A, Serum albumin level and nosocomial pneumonia in stroke patients. Eur J Neurol 13 (2006): 299–301. 16618350 10.1111/j.1468-1331.2006.01210.x

[28] Wang C, Deng L, Qiu S, Serum Albumin Is Negatively Associated with Hemorrhagic Transformation in Acute Ischemic Stroke Patients. Cerebrovasc Dis 47 (2019): 88–94. 30897566 10.1159/000498855

[29] Belayev L, Zhao W, Pattany PM, Diffusion-weighted magnetic resonance imaging confirms marked neuroprotective efficacy of albumin therapy in focal cerebral ischemia. Stroke 29 (1998): 2587–2599. 9836772 10.1161/01.str.29.12.2587

[30] Belayev L, Liu Y, Zhao W, Human albumin therapy of acute ischemic stroke: marked neuroprotective efficacy at moderate doses and with a broad therapeutic window. Stroke 32 (2001): 553–560. 11157196 10.1161/01.str.32.2.553

[31] Seners P, Turc G, Oppenheim C, Incidence, causes and predictors of neurological deterioration occurring within 24 h following acute ischaemic stroke: a systematic review with pathophysiological implications. J Neurol Neurosurg Psychiatry 86 (2015): 87–94. 24970907 10.1136/jnnp-2014-308327

[32] Helleberg BH, Ellekjaer H, Indredavik B. Outcomes after Early Neurological Deterioration and Transitory Deterioration in Acute Ischemic Stroke Patients. Cerebrovasc Dis 42 (2016): 378–386. 27351585 10.1159/000447130

